# Management of Partial Edentulism Using Nonrigid Connectors as a Treatment Modality: A Case Report

**DOI:** 10.7759/cureus.7790

**Published:** 2020-04-23

**Authors:** Sheela Sivakumar

**Affiliations:** 1 Prosthodontics, Dr. M.G.R. Educational and Research Institute, Chennai, IND

**Keywords:** pier abutment, key and keyway, non rigid connectors

## Abstract

The most frequently encountered clinical situation, either in the maxillary or mandibular arch, is of a missing first premolar and first molar, where the canine and the second molar are known as terminal abutments and second premolar is a pier abutment. This clinical situation poses challenge to prosthodontist in rehabilitation phase. It has been postulated that terminal abutment has a rocking movement when in function, whereas pier abutment acts as a fulcrum. This will lead to debonding of the less retentive terminal retainer. In order to overcome this, utilization of nonrigid connectors has been advised. This paper presents a clinical case report which describes incorporation of nonrigid connector to rehabilitate pier abutment case.

## Introduction

There are some important variables that are responsible for the longevity of a fixed partial denture (FPD) and its abutments such as proper occlusion, span length, bone loss surrounding the abutment, and the quality of the periodontium [1]. Excessive flexing of the long-span FPD will lead to material failure or unfavorable prosthesis failure. The occlusal forces are transmitted to the supporting structures through the pontic, connectors, and retainers in FPD prosthesis [2].

A FPD with the pontic rigidly fixed to the retainer provides adequate strength and stability to the prosthesis and also minimizes the stresses associated with the restoration. But if an edentulous space occurs on both sides of a tooth, creating a pier abutment then physiologic tooth movement, arch position of the abutment, and a difference in the retentive capacity of the retainers can make a five-unit FPD a less than ideal for the treatment [2].

Biomechanical factors such as overload, leverage, torque, and flexing induce abnormal stress concentration in a FPD [3]. Stress concentration is found in the connectors of the prosthesis and in the cervical dentin area near the edentulous ridge. This factor further plays an important role in the failure of the long-span FPD. Connectors are that portion of the FPDs that unite the retainers and the pontics. They are of two types, rigid connectors and nonrigid connectors [3].

Rigid connectors are made by different procedures such as casting, soldering, and welding. The cast connectors are to be properly shaped in wax patterns. The connector that permits limited movement between the otherwise, independent members of the FPDs is the nonrigid connector. The nonrigid connector could be made by incorporation of prefabricated inserts, by use of a custom-milling machine, or by use of the prefabricated plastic patterns [4]. The purpose of this article is to summarize the treatment approaches in cases of pier abutment to minimize the effect of forces in long-span bridges. The treatment options in case of pier abutment are implant in edentulous spaces or FPD with nonrigid connectors, using precision and semi-precision attachments.

## Case presentation

**Figure 1:**
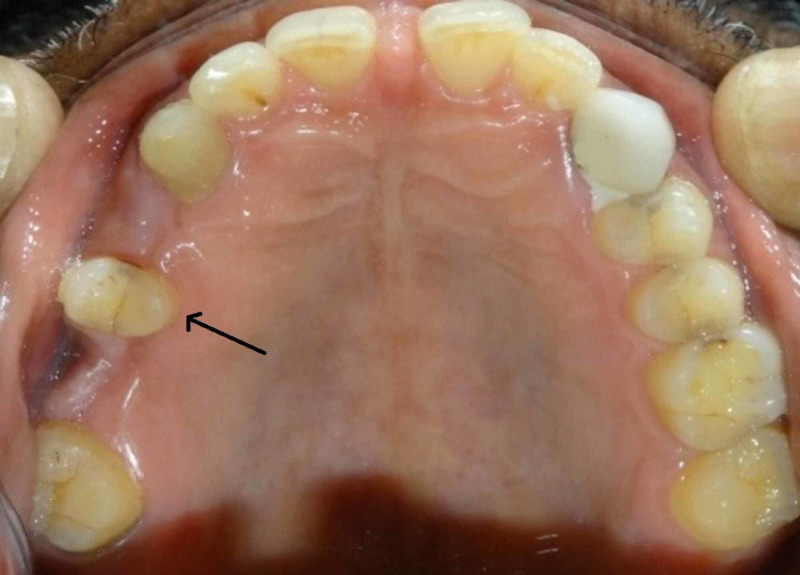
Intraoral photograph with arrow indicating the pier abutment.

A 43-year-old male patient reported to the Department of Prosthodontics in our dental college in Chennai, India with a chief complaint of missing teeth in the right upper back tooth region for a duration of six months. He also complained of difficulty in mastication as well as esthetic problem. There is no relevant past medical history and past dental history revealed that the patient had undergone extraction of the badly carious tooth in left maxillary first premolar and first molar three months back. On intraoral examination we found missing left maxillary first premolar and first molar with second premolar acting as a pier abutment (Figure 1).

**Figure 2:**
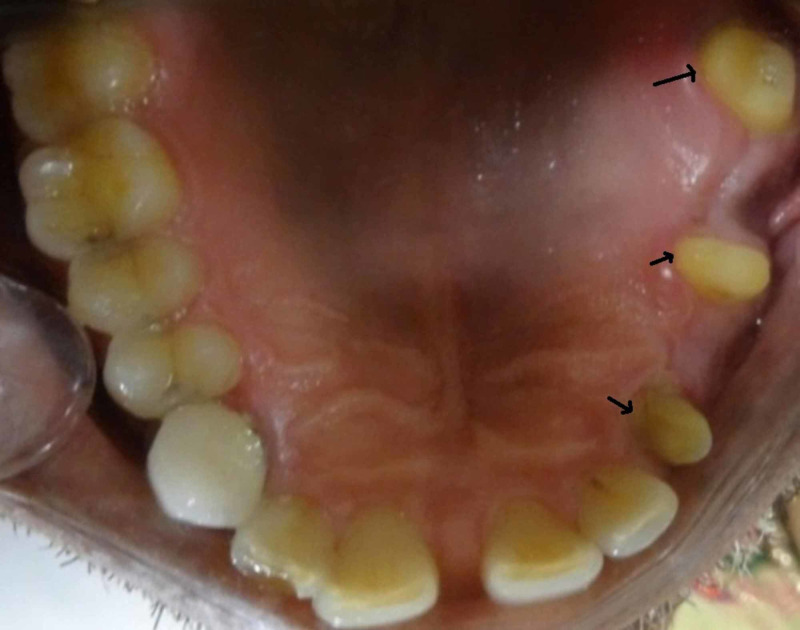
Tooth preparation of maxillary left canine, second premolar, and second molar.

Different treatment options were discussed with the patient, and with the patient’s consent, we decided to rehabilitate the edentulous space using nonrigid connector in the distal aspect of the pier abutment. The following clinical steps were carried out for oral rehabilitation. The patient preferred metal ceramic restoration. Hence tooth preparation was done on left maxillary canine, second premolar, and second molar (Figure 2) with equigingival margins and shoulder finish line incorporated in the preparation for better outcome of the restoration.

The gingival retraction was done with gingival retraction cord and final impression was made using elastomeric impression material with two-stage putty wash technique. Interocclusal record was made using bite registration material to obtain good occlusion of the patient. Provisional temporary restoration was given using tooth colored auto polymerizing resin and was cemented using noneugenol temporary cement. Type IV dental stone was used to pour cast. After the material was completely set, it was retrieved and die cutting was done and the die pins were placed. Master cast was then mounted on an articulator with the help of interocclusal record.

**Figure 3:**
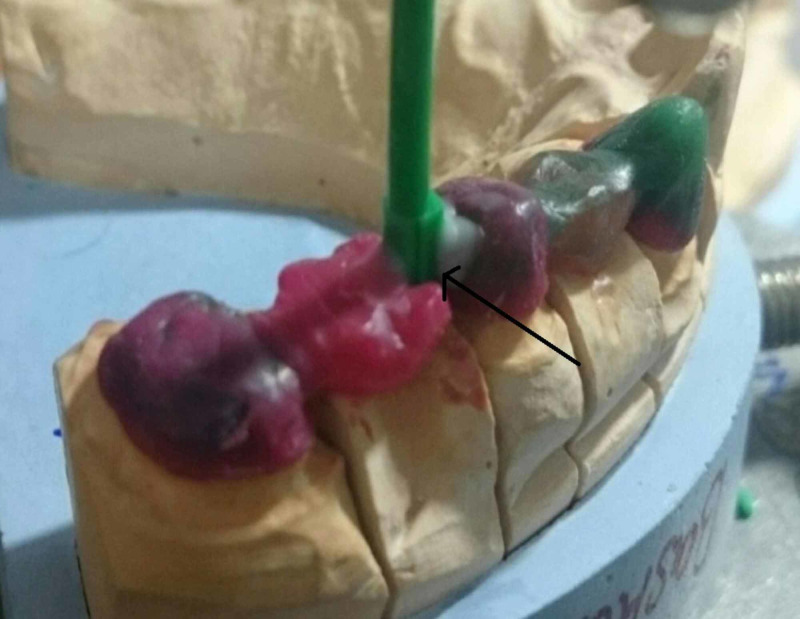
Wax pattern in maxillary left canine, first and second premolar, first and second molar with arrow mark representing prefabricated attachment.

Wax pattern (Figure 3) was fabricated in the laboratory on the maxillary left canine, first premolar, and second premolar with a female prefabricated attachment on the distal aspect of pier abutment. The pattern is invested, burned out, and cast. After the casting has been cleaned and pickled, any part of the keyway portion of the attachment that protrudes above the occlusal surface is carefully cut off.

 

Surveying was done using Ney surveyor which helps in checking the position and parallelism of female and male component placed within the contours of the pier abutment. Male pattern was later retrieved from the female pattern, so that the female pattern is free of wax in the recess area. After the casting is recovered from the investment, the mandrel and any excess on the top portion of the key are carefully reduced so the key and keyway were in flush with each other.

**Figure 4:**
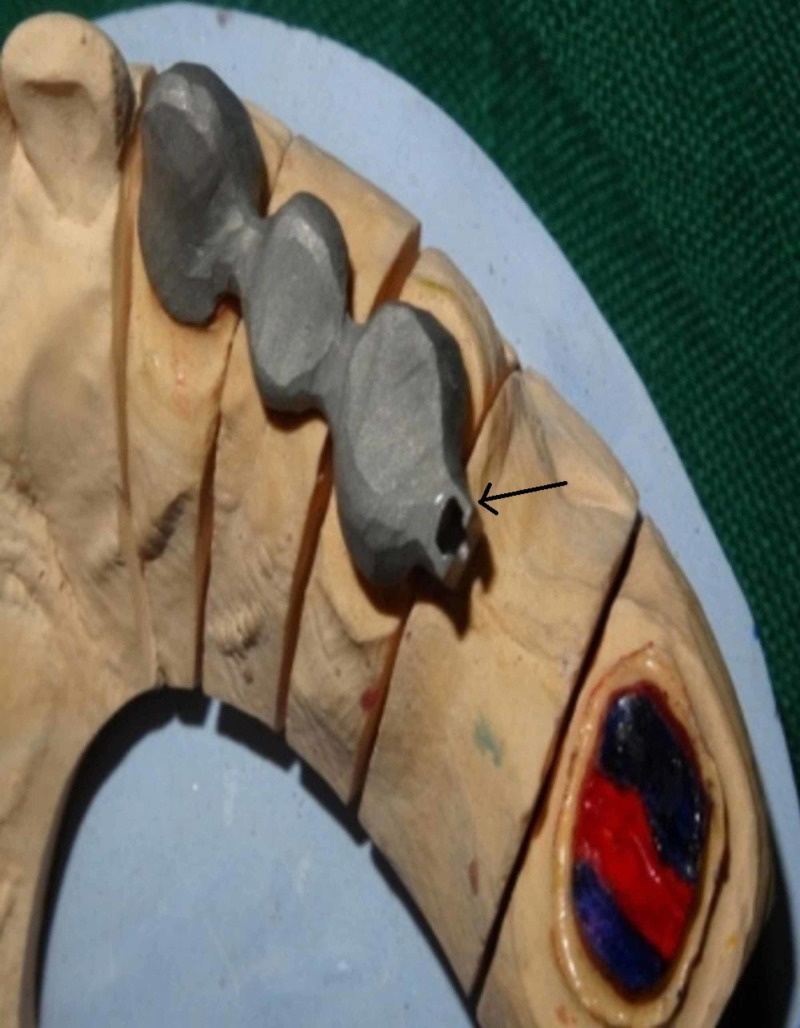
Casted metal in the anterior segment with arrow representing the female component (Keyway or Mortise).

After casting, metal trimming was done and extension of female pattern was cut accordingly. Later metal try-in of the anterior segment (female part) was done in the laboratory (Figure 4) in the working cast to check the marginal fit of the casted metal units.

**Figure 5:**
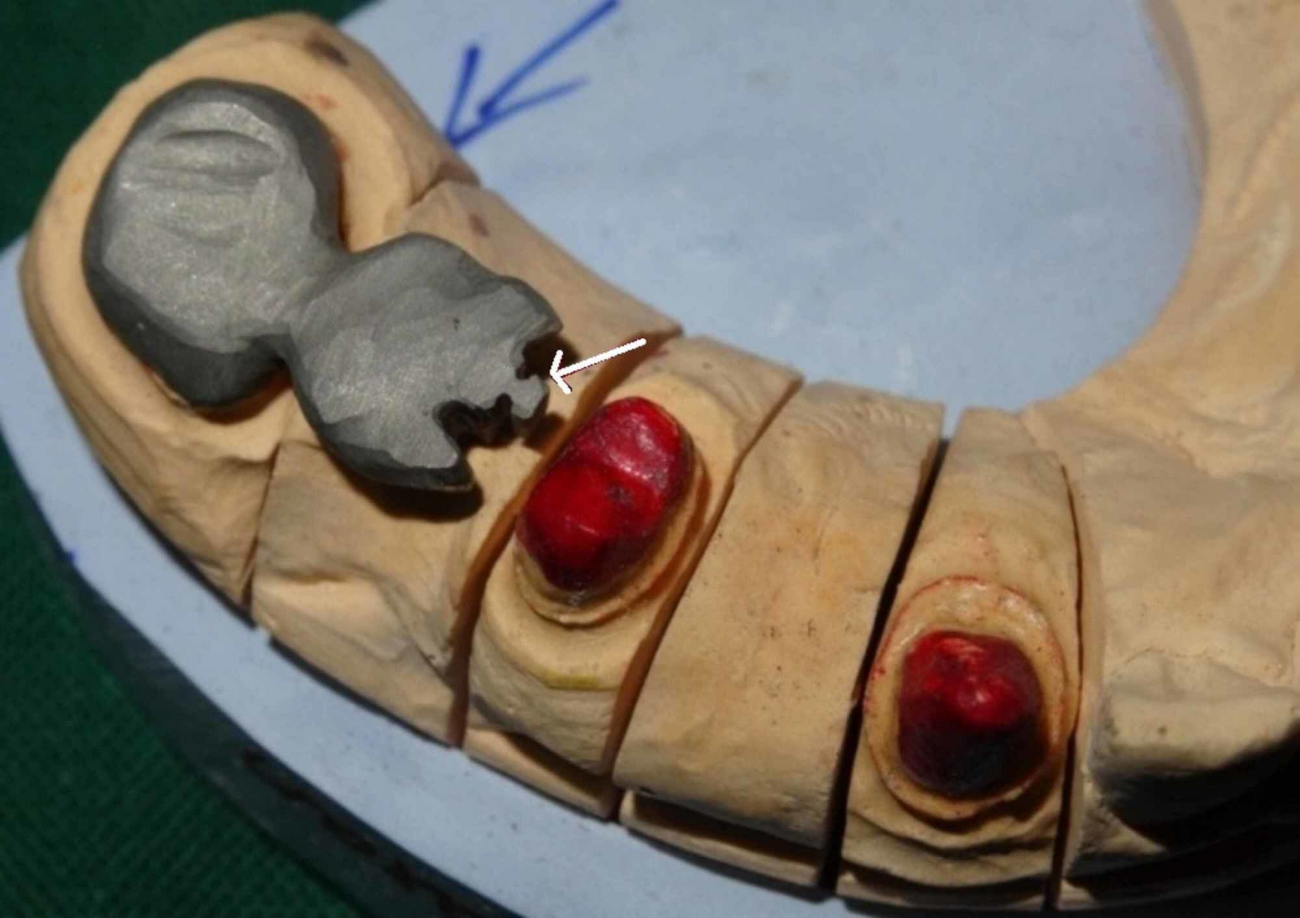
Casted metal in the posterior segment with arrow representing the male component (Key or Tenon).

The posterior segment (male pattern) was seated in the casted female portion, then wax pattern was fabricated for left maxillary first molar and second molar. Similar casting procedures were carried out (Figure 5) and the marginal fit was checked in the laboratory for the posterior segment also.

**Figure 6:**
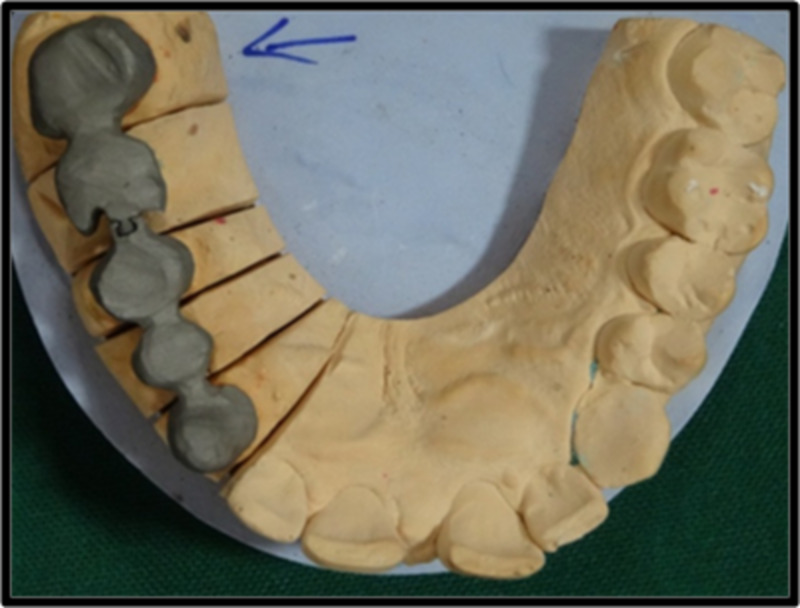
Completed male and female metal units.

Both male and female portions were placed and the metal fit was checked in the lab (Figure 6).

**Figure 7:**
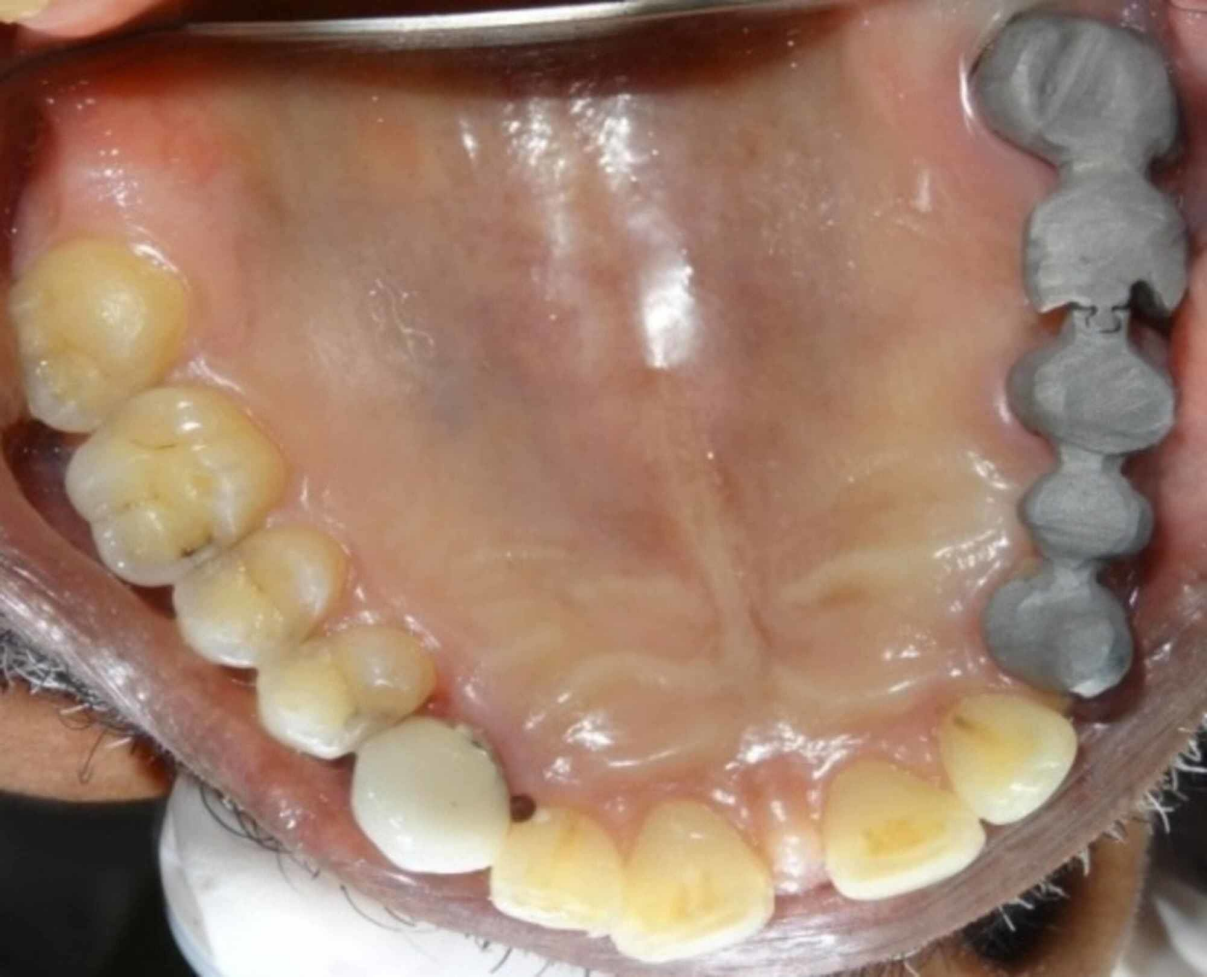
Intraoral metal try-in.

Metal try-in of both anterior and posterior segment was done clinically to verify properly the marginal fit of the restoration (Figure 7). Then it was subjected to ceramization after shade selection.

**Figure 8:**
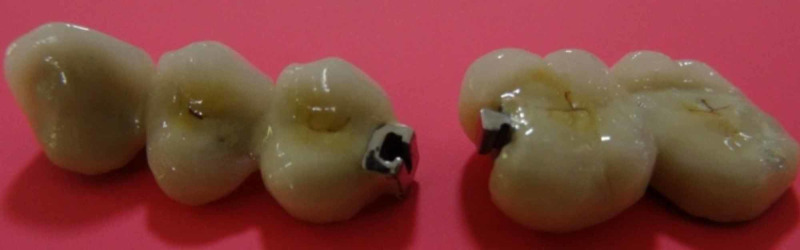
Completed five-unit FPD. FPD, fixed partial denture

**Figure 9:**
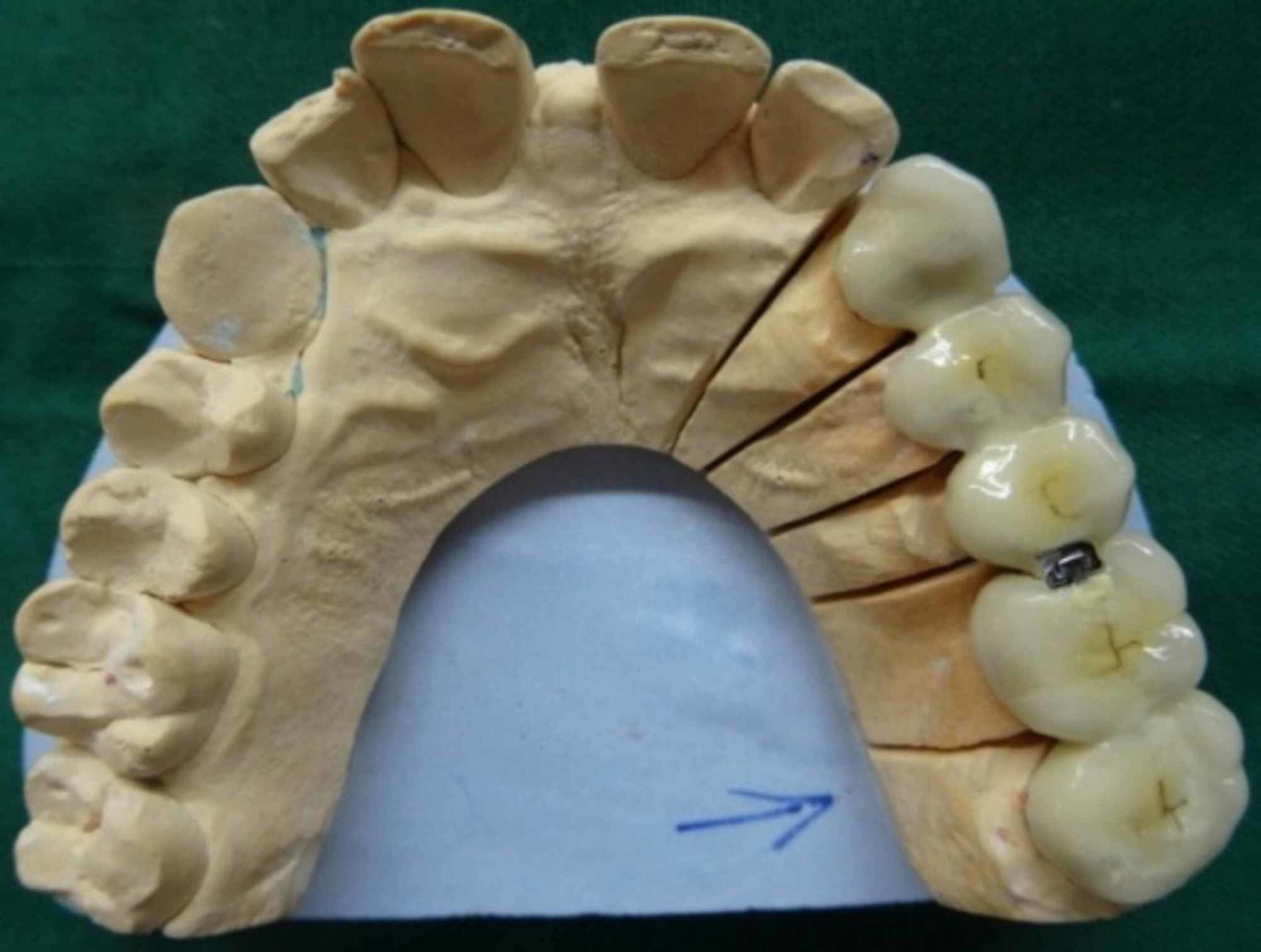
Male and female portion assembled with nonrigid connector.

Completed five-unit FPD is shown in Figure 8. Anterior segment with female portion (keyway mortise) and posterior segment with male portion (key tenon) were assembled together in the working cast completing the laboratory procedure (Figure 9).

**Figure 10:**
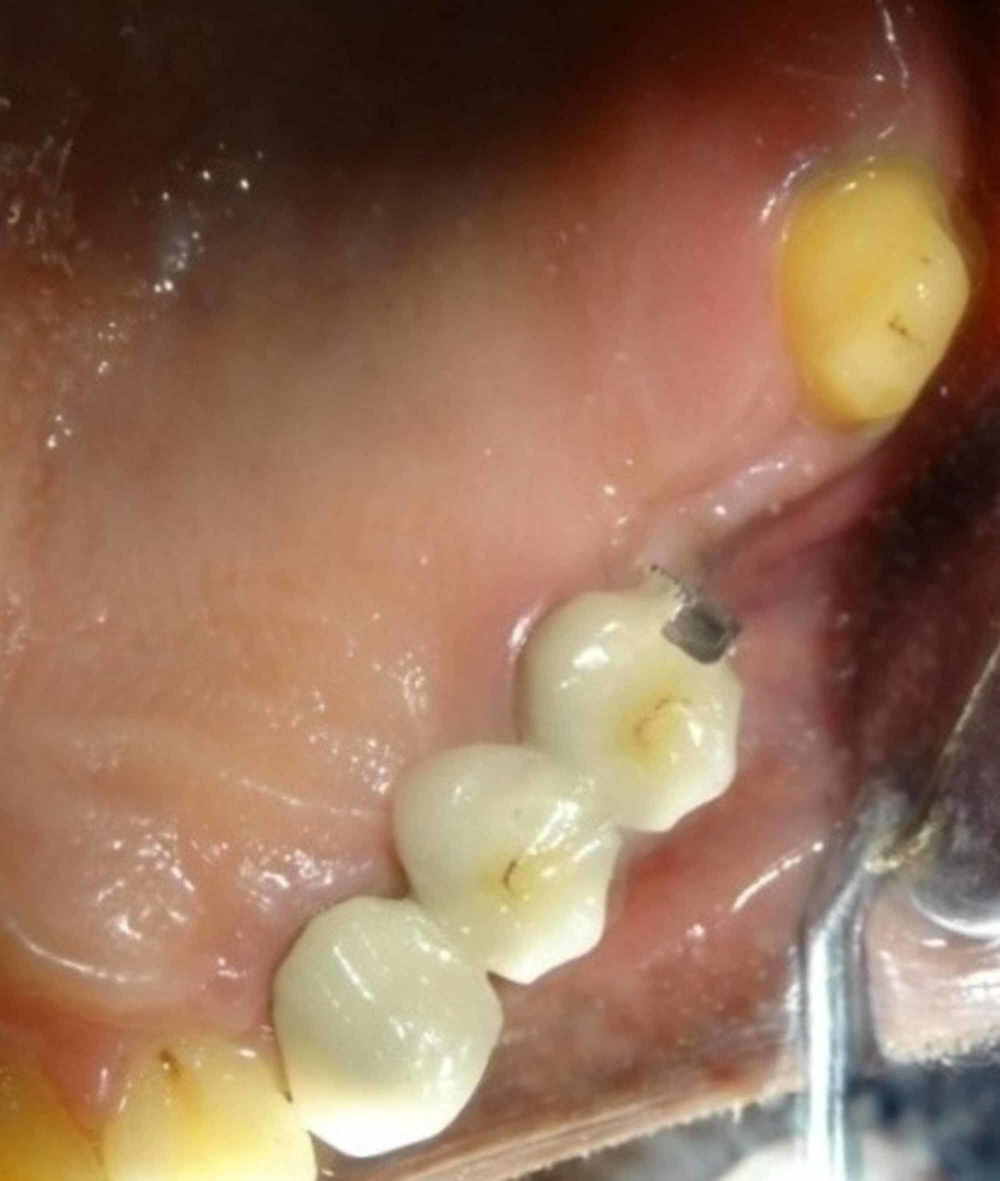
Intraoral cementation of anterior segment.

**Figure 11:**
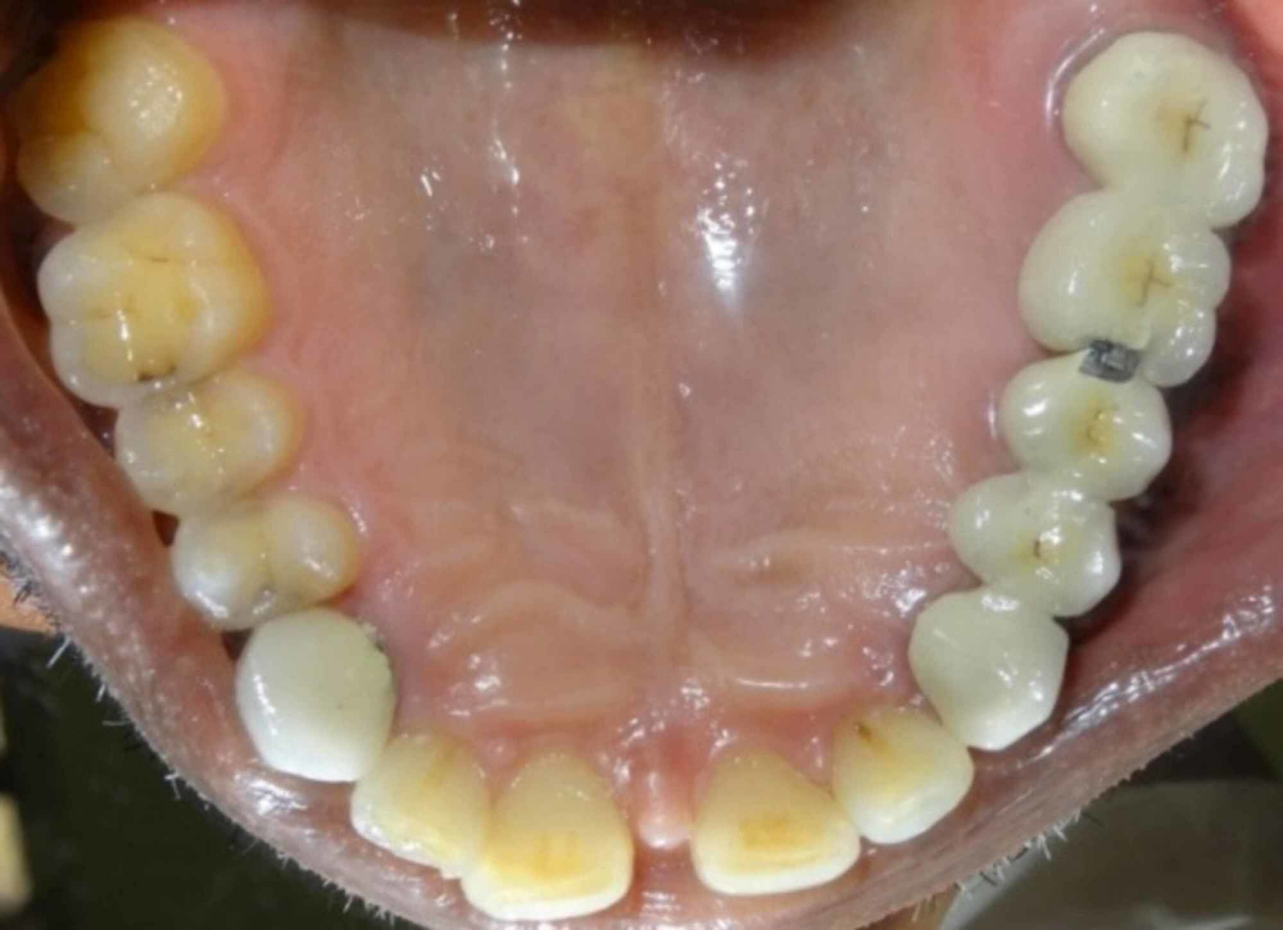
Completed cementation of five-unit bridge.

Clinically, three-unit segment containing the left maxillary canine, first premolar, and second premolar (pier abutment) with keyway on its distal aspect was cemented (Figure 10) first followed by cementation of posterior two-unit segment containing left maxillary first molar and second molar with key on the mesial contour of the first molar (Figure 11). Cementation was done using Type I glass ionomer cement.

**Figure 12:**
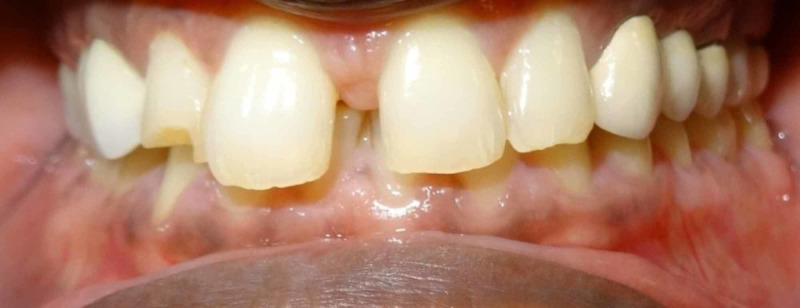
Postclinical photograph.

Then the excess cement is removed and the occlusion was verified using articulating paper. The patient was advised to use dental floss regularly to maintain good oral hygiene and interdental brush if needed. The patient was reviewed after one week (Figure 12) to further evaluate the occlusion and oral hygiene and the restoration was a successful one.

## Discussion

The existence of pier abutment promotes a fulcrum-like situation that can cause the weakest of the terminal abutments to fail and may cause the intrusion of a pier abutment [5]. Shear stresses are concentrated on the supporting bone and not on the connector in a stress breaker; hence the need for a stress breaker in pier abutment on both ends of the nonrigid connector is recommended. A stress breaker minimizes mesio-distal torquing of abutments and permits them to move independently [6].

The rigid and nonrigid connectors can increase the lifespan of an abutment in the five-unit FPD as it transfers less stress on the abutments. Further it allows physiologic tooth movement and eliminates any hindrance against a fixed restoration with all rigid connectors [7]. FPDs are standard treatment options before the advent of implant therapy. The long-term survival of FPDs has been reported to be 87% at 10 years and 69% at 15 years [8]. Critical factors that are responsible for failures included nonvital anterior abutments and pier abutments [9]. Hence, in such cases, dental implant can be a better alternative in such patients provided there is good bone support and the patient is financially affordable.

Nonrigid connectors are indicated in places where there is existence of pier abutment, and in cases like misaligned abutment where the use of intracoral attachments as connectors is indicated [5]. It is also used in long-span prosthesis where there are chances of distortion and shrinkage of porcelain which leads to ill-fitting prosthesis. Another important indication is in cases of mobile teeth which requires splinting as a treatment option where interlocks are used for stabilization of the units. When this reported case like clinical situation occurs in mandible, nonrigid connectors which are indicated as mandible undergoes flexing movement in a mediolateral direction during opening and closing of jaws.

 Nonrigid connectors are contraindicated when there is significant mobility in abutment teeth where splinting also cannot produce good prognosis [10]. It is also contraindicated when the edentulous span involves more than one tooth. Nonrigid connectors were also not indicated in cases where there are discrepancies and interferences with regard to occlusion and occlusal forces.

According to literature there are four types of nonrigid connectors such as Tenon-Mortise type connectors, Cross-pin and Wing type connector, Split type connector, and Loop type connector. The most widely used type is Tenon-Mortise type where accurate position of the Mortise is technique sensitive as it has to establish the parallelism for accurate path of withdrawal of a distal retainer. Selection of the right type of connector makes the difference between success and failure of the restoration [11]. Under conditions of vertical loading, the rigid FPD design does not permit independent response by either abutment. The nonrigid FPD design allows the abutments some independence in response to vertical loading [12]. Some of the researchers employed quasi three-dimensional photoelastic stress analysis where they concluded that some amount of stress and occlusal displacement were obvious on continuous loading of the FPDs [13]. Savion et al. in 2006 did a study using a mathematical model where he concluded that debonding may occur in the anterior abutment, but not due to the teetering of the FPD around the pier abutment [14].

Though nonrigid connectors have the advantage of minimizing the torquing forces and shear stresses, they have a disadvantage also. They involve more tooth reduction clinically in order to facilitate engagement of male and female components. It is more technique sensitive and requires precision in work. When there is no occlusal stability, lifting of key from key is also reported.

 Shillinburg and Fischer in 1973 suggested that patrix should be prepared within the contours of the retainer and matrix attached to the distal of pontic. The pier abutment should have the keyway of the connector in the distal and the key should be placed on the mesial of the distal most pontic. If the keyway of the connector is placed on the distal side of the pier abutment, mesial movements seats the key into the keyway [15]. Oruc et al. in 2008 attempted a study using finite element method and observed that the distal area of pier abutment has minimum stress concentration which supported the placement of nonrigid connectors at the distal region of premolar [16]. Markley suggested that the stress of the movement of one tooth prying against the others was eliminated much as a broken-stress joint frees a fixed bridge from destructive strain [17].

Gill in 1952 suggested that each tooth must function both as an individual and as a part of a collective unit which makes each tooth an important factor in the function of the entire mouth [18]. Adams in 1956 suggested placing one nonrigid connector at the distal side of pier and also at the distal of the anterior retainer if needed depending on the clinical situation [19]. Nimushara et al. in 1999 studied the stress transfer patterns with variable implant support and simulated natural teeth through rigid and nonrigid connection under simulated functional loads and concluded that the rigid connector in particular situation caused only slightly higher stresses in the supporting structure and demonstrated more widespread stress transfer. Misch suggested that bone and soft tissue consideration is important when planning for a long-span bridge. For a natural pier abutment two implants can be one of the treatment option where stress breaker is not indicated [20]. By such treatment option, we are completely avoiding the load and fulcrum-like situation associated with the pier abutment. However, implants can only be placed after complete medical, clinical, and radiological evaluation. In cases where implants cannot be placed due to compromised conditions, the nonrigid connectors are advocated. Hence precision and semi-precision attachments provide room for slight movements which prevent loading of the pier abutment created due to the fulcrum-like situation and increase the lifespan of five-unit FPD.

## Conclusions

Thus the factors such as physiologic tooth movement, arch position of the abutments, and retentive capacity of retainers make the rigid connectors a less ideal plan of treatment in cases involving pier abutments particularly five-unit bridges. Broken-stress measures serve as “safety valves” against the tremendous leverage forces created by the rigid attachment to two or more teeth. The size, shape, and type of connectors also play a key role in future success of a FPD. The employment of nonrigid connector increases the life of the restoration as it minimizes the stresses on the abutments. The stress distributions and values of a FPD and a pier abutment are affected by the location of nonrigid connector. Apart from the advantages of nonrigid connectors, the increased laboratory time and expense should be ignored while considering the augmented life of the restoration.

## References

[1] (2002). Contemporary Fixed Prosthodontics-E-Book, 3rd Edn.

[2] Yaqoob A, Rasheed N, Ashraf J, Yaqub G (2014). Non-rigid semi precision connectors for FPD. Dent Med Res.

[3] Mattoo K, Brar A, Goswami R (2014). Elucidating the problem of pier abutment through the use of a fixed movable prosthesis - a clinical case report. Int J Dent Sci Res.

[4] Sherring-Lucas M, Martin P (1994). Attachments for Prosthetic Dentistry. Introduction and Application. Implant Dentistry.

[5] Badwaik PV, Pakhan AJ (2005). Non-rigid connectors in fixed prosthodontics: current concepts with a case report. J Indian Prosthodont Soc.

[6] Sudhir N, Taruna M, Suchita T, Bharat M (2011). Indigenously fabricated non-rigid connector for a pier abutment. Indian J Dent Advance.

[7] Banerjee S, Khongshei A, Gupta T, Banerjee A (2011). Non-rigid connector: the wand to allay the stresses on abutment. Contemp Clin Dent.

[8] Walton TR (2002). An up to 15-year longitudinal study of 515 metal-ceramic FPDs: part 1. Outcome. Int J Prosthodont.

[9] Jivraj S, Chee W (2006). Rationale for dental implants. Br Dent J.

[10] Shillingburg HT, Sather DA, Wilson EL, Cain JR, Mitchell DL, Blanco LJ (2012). Fundamentals of Fixed Prosthodontics. Fundamentals of fixed prosthodontics. Chicago: Quintessence Publishing Co.

[11] Malone WF, Tylman SD, Koth DL (1989). Tylman's Theory and Practice of Fixed Prosthodontics. Ishiyaku EuroAmerica, Incorporated.

[12] Sutherland JK, Holland GA, Sluder TB, White JT (1980). A photoelastic analysis of the stress distribution in bone supporting fixed partial dentures of rigid and nonrigid design. J Prosth Dent.

[13] Standlee JP, Caputo AA (1988). Load transfer by fixed partial dentures with three abutments. Quintessence Int.

[14] Savion I, Saucier CL, Rues S, Sadan A, Blatz M (2006). The pier abutment: a review of the literature and a suggested mathematical model. Quintessence Int.

[15] Akulwar RS, Kodgi A (2014). Non-rigid connector for managing pier abutment in FPD: a case report. J Clin Diagn Res.

[16] Oruc S, Eraslan O, Tukay HA, Atay A (2008). Stress analysis of effects of nonrigid connectors on fixed partial dentures with pier abutments. J Prosth Dent.

[17] Markley MR (1951). Broken-stress principle and design in fixed bridge prosthesis. J Prosth Dent.

[18] Gill JR (1952). Treatment planning for mouth rehabilitation. J Prosth Dent.

[19] Adams JD (1956). Planning posterior bridges. J Am Dent Assoc.

[20] Misch CE (2008). Contemporary Implant Dentistry, 3rd edition. Implant Dent.

